# “The elephant in the room;” a qualitative study of perinatal fears in opioid use disorder treatment in Southern Appalachia

**DOI:** 10.1186/s12884-021-03596-w

**Published:** 2021-02-17

**Authors:** Catherine Leiner, Tamara Cody, Nathan Mullins, Melinda Ramage, Bayla M. M. Ostrach

**Affiliations:** 1 UNC School of Medicine, 321 S Columbia St, Chapel Hill, NC 27516321 USA; 2 Mountain Area Health Education Center, 119 Hendersonville Rd, Asheville, NC 28803 USA; 3 Department of Research, UNC Health Sciences at MAHEC, 121 Hendersonville Road, Asheville, NC 28803 USA

**Keywords:** Opioid use disorder, Perinatal substance use treatment, Medication assisted treatment, Social services, CAPTA laws

## Abstract

**Background:**

Diagnoses of perinatal opioid use disorder (OUD) continue to rise in the United States. Patients and providers report obstacles to OUD treatment access. Difficulties include legal ambiguity related to Social Services notification requirements following a birth to people using opioids or in medication-assisted treatment for OUD.

**Methods:**

Through semi-structured interviews, participant-observation, and a focus group conducted in a mostly rural, region of the Southern United States (where perinatal OUD is more prevalent), patients’ and providers’ perspectives about perinatal substance use treatment were initially sought for a larger study. The findings presented here are from a subset analysis of patients’ experiences and perspectives. Following ethics review and exemption determination, a total of 27 patient participants were opportunistically, convenience, and/or purposively sampled and recruited to participate in interviews and/or a focus group. Data were analyzed using modified Grounded Theory.

**Results:**

When asked about overall experiences with and barriers to accessing perinatal substance use treatment, 11 of 27 participants reported concerns about Social Services involvement resulting from disclosure of their substance use during pregnancy. In the subset analysis, prevalent themes were *Fears of Social Services Involvement*, *Preparation for Delivery*, and *Providers Addressing Fears*.

**Conclusions:**

Perinatal OUD patients may seek substance use treatment with existing fears of Social Services involvement. Patients appreciate providers’ efforts to prepare them for this potential reality. Providers should become aware of how their own hospital systems, counties, states, and countries interpret laws governing notification requirements. By becoming aware of patients’ fears, providers can be ready to discuss the implications of Social Services involvement, promote patient-centered decision-making, and increase trust.

**Supplementary Information:**

The online version contains supplementary material available at 10.1186/s12884-021-03596-w.

## Background

Opioid use disorder (OUD) diagnoses have been increasing rapidly in the United States, particularly among patients biologically capable of pregnancy [1–3]. Rates of opioid use disorder (OUD) specifically detected at delivery increased fourfold in the United States over the past decade [4] and pose significant perinatal health risks for the dyad composed of the neonate and the person that delivered it (sometimes referred to as the mother-baby dyad, though we recognize that transmen and other non-cisgender people also deliver babies) [3, 5, 6]. Perinatal OUD is thus a particular area of focus within obstetrical care [3, 7]. Some health concerns related to OUD in pregnancy and postpartum include increased risks for fetal growth restriction, preterm labor, and neonatal abstinence syndrome (NAS); newly referred to as neonatal opioid withdrawal syndrome [5, 8]. Fortunately, treatments for perinatal OUD exist that have been proven safe and efficacious and are recommended by major medical associations.

However, a pregnant person with OUD is often reluctant to seek prenatal care according to recommended guidelines [9–11], in part because of the stigmatized nature of substance use during pregnancy and stigma associated with medication-assisted treatment (MAT), specifically [9, 10, 12]. Laws are increasingly in place throughout the United States that penalize substance use during pregnancy [11, 13]. These include mandatory notifications or reporting requirements that are shown to deter pregnant people from seeking either prenatal care or substance use treatment [11, 14, 15]. As noted by O’Rourke-Suchoff et al. [16] and others [17–19], such laws and requirements also threaten perinatal OUD patients’ trust in care providers.

Literature published to date about pregnant and postpartum OUD patients’ perceptions of Social Services/child services largely documents people’s fears; the deterrent effect on seeking care; and the threats posed to relationships with care providers. In one such study of barriers to care in the Midwestern U.S. [18], women who used substances during their most recent pregnancy mentioned a desire to be honest with providers about their substance use. However, they reported the outcome of such disclosure was not necessarily good. The researcher concluded it is likely less risky for women to disclose use when the relationship with their established provider is a trusting one. Calling for more research on the topic, the author concluded that substance use treatment providers should offer information to help patients be prepared for what might occur if Social Services become involved.

In recent research with postpartum SUD patients in rural areas, participants emphasized the importance of relationships with their care providers and a desire for these providers to understand their experiences [20]. Reinforcing the findings of an earlier study in the Northeastern U.S. [21], women with OUD in the same region interviewed about their labor and delivery experiences described their state’s mandatory child protective services reporting requirement as threatening. They reported the looming possibility of child removal negatively impacted relationships with their care providers [16]. The authors of the more recent study recommended education about the role of child protective services – for patients and providers.

Yet little existing literature illuminates how pregnant and postpartum people who sought and accessed OUD treatment as part of prenatal care perceived the spectre of government surveillance and involvement in their parenting. Research also does not explore how providers addressed and alleviated such concerns. The purpose of this article is to facilitate greater understanding of perinatal OUD patients’ fears, concerns, needs, and priorities and provide lessons for reproductive health and substance use treatment providers.

### Clinical care for perinatal OUD

All major medical associations in the United States recommend treating OUD with medication-assisted treatment (MAT); including during pregnancy, within comprehensive perinatal substance use treatment settings [2, 3, 7, 22, 23]. Comprehensive perinatal substance use treatment includes OB/Gyn care, clinically appropriate treatment for the diagnosed substance use disorder, behavioral health support, and linkage to community-based substance supportive services [7, 22]. For the purposes of this article, and in the U.S. outpatient obstetric setting, MAT refers to the use of medically prescribed buprenorphine or buprenorphine-naloxone products used to reduce or eliminate withdrawal symptoms and cravings associated with OUD. Provision of MAT, specifically buprenorphine products, reduces perinatal complications and risk of return to use and/or overdose during pregnancy [5]. A growing body of literature demonstrates the greater safety and efficacy of buprenorphine products (compared to either methadone or no treatment) for infant outcomes [24–26].

### Access to medication-assisted treatment

Though safe, effective, and recommended for OUD treatment, access to MAT is uneven and geographically stratified. There is significantly less access to MAT in the U.S. South^1^ [27], despite higher rates of OUD and previous research documenting the highest national rates of opioid prescribing among people capable of pregnancy [28, 29]. Factors contributing to the lack of MAT access in the U.S. South include shortage of prescribers; less health insurance coverage through Medicaid (state-funded coverage based on income – the extent of eligibility and coverage varies by state, and there is much less access in the South); and overall greater barriers to care [28–30]. Less Medicaid coverage is a particular barrier for this population as most deliveries in the U.S. are by people on Medicaid [31]; many pregnant and immediately postpartum people with OUD rely on public insurance programs, namely Medicaid -- the only potential public health coverage for most low-income U.S. residents [32, 33]. As a result, in the many U.S. states where Medicaid coverage ends shortly after delivery, OUD treatment is also largely limited to pregnancy and may end abruptly for new parents. Compounded with gaps in financial coverage for treatment, patients in rural settings also struggle with limited transportation and increased substance use stigma [20]. Cisgendered women of color, pregnant people in poverty, and people from other marginalized communities all encounter more obstacles to substance use treatment, especially MAT [34–36]. Obstacles to access are compounded by drug user stigmatization [37], and stigma toward MAT [9, 10] – the latter particularly documented in the region where this study was conducted.

### Provider barriers and awareness of notification requirements

Clinicians also face barriers providing perinatal MAT in the U.S. Offering such care is complicated by federal Child Abuse Prevention and Treatment Act (CAPTA) laws, interpretations, and enforcement of which vary by state. The federal CAPTA law stipulates that *policies and procedures to address needs of infants born with and/or identified as being affected by prenatal drug exposure or withdrawal symptoms* include a *requirement that health care providers involved in the delivery or care or such infants notify child protective services systems of the occurrence of such conditions*. The law mandates *referrals to* [municipal or state] *child protective services systems* [38]... . Confusion persists for providers as to the definition of terms in the above clauses of the law, such as “*affected by*” [exposure]; and over which deliveries require simply a notification versus which should prompt a formal report to child protective services (CPS) which may trigger immediate removal of an infant from the parent to whom it was just born. The lack of clear definitions of “affected by” and “exposure” in the federal CAPTA law, or in state and local versions of it, permits hospitals to develop their own criteria for reporting. In many instances, without corresponding referrals to agencies or providers who may offer supportive care to families.

Because of this confusion, there is significant variability in circumstances that result in a notification being made to CPS. Within the realm of OUD treatment, it ranges from infants receiving prescription treatment for opioid withdrawal symptoms, to all infants born exposed to MOUD even when the person to whom they were delivered used it as prescribed by a licensed provider. Notification procedures thus vary institution by institution, county by county, and state by state [39]. Understandably, providers may not be aware of the mandated notification or how their healthcare institution, county, or state interprets these laws [17].

In this article we argue that, as shown in earlier studies [16, 18, 21] perinatal OUD patients are keenly aware and afraid of the potential for Social Services to become involved at the time of delivery, even if they do not understand the CAPTA law. In addition, we suspect gaps in awareness of CAPTA between providers and patients may affect providers’ ability to understand their perinatal patients’ fears, needs, and concerns. Such lack of understanding can result in missed opportunities to support perinatal patients in substance use treatment who fear child removal, identified in earlier studies cited above. In some settings, both hospitals and outpatient care, specific attempts have been undertaken to raise awareness about perinatal substance use, CAPTA laws, and notification requirements. One such training program enhanced provider knowledge of relevant laws; increased referrals to evidence-based treatment programs, and reduced self-reported provider stigma toward women using substances [40].

### Perinatal substance use exposure education

In the program studied for this article, a perinatal substance use exposure educator was an embedded community partner working as part of the provider team. The perinatal substance use exposure educator (PSE) teaches perinatal OUD patients what to expect at the time of delivery, in relation to CAPTA and local Social Services notification. In this program, PSE consultation is recommended early in the third trimester in preparation for delivery but the PSE member could meet with a patient at any time in pregnancy if a patient reported particular concerns, for example based on having experienced child removal in a previous pregnancy (as was not uncommon).

A PSE can review with a patient what to expect at delivery concerning neonatal monitoring, such as hospital staff members’ routine use of Finnegan scores^2^ to assess symptoms of NAS/NOWS [41], recommended length of stay (LOS) for the newborn, and additional wrap-around services that may be initiated when a neonate is identified as having had prenatal exposure to substances. At the time, in the program we studied, the PSE was an embedded social worker from the Neonatal Intensive Care Unit (NICU) of a hospital in the region, available on-site in the perinatal substance use treatment clinic on specific days of the week. A trained social worker, the PSE, a trained social worker identified patients whom the hospital would recognize as having OUD and being on MAT and informed them about what the process of delivering at the hospital would entail – including the potential for a notification to Social Services or report to child protective services.

At the time of this research, the clinic where we conducted research addressed CAPTA Law and automatic notification requirements by having the PSE integrated as part of the comprehensive clinic team. The PSE was available to educate patients on current guidelines for how notifications to Social Services would be made post-delivery, based on state policies at the time. The PSE informed patients on who they would interact with at the hospital or what would happen if Social Services become involved. When loss of custody was a concern, the PSE discussed options for if a newborn were to be placed outside of the pregnant person’s care – such as with a relative the parent designates (kinship provider) or with a ‘temporary safety provider’ designated by children’s services.^3^ These conversations could be time-consuming and unfold over several appointments.

In this article, we present perinatal OUD patients’ perceptions of and experiences in a comprehensive substance use treatment program, with a particular focus on their discussions of the potential for Social Services involvement. Our findings include lessons offered in patients’ descriptions of how program staff addressed their fears.

## Methods

### Study site

This article presents a sub-analysis of specific themes emerging from a larger qualitative study investigating patient and provider experiences in a perinatal substance use treatment program in Southern Appalachia [42]; a program housed within a high-risk obstetrics and gynecology practice serving a large, mostly rural region. The perinatal substance use treatment program serves pregnant and postpartum patients from a large region; it is one of the only programs of its kind in this part of the country. It also serves patients with many kinds of substance use disorders, not only OUD. Those patients diagnosed with OUD report using a range of opioids, including diverted prescription pills, injected heroin (with and without fentanyl), smoked heroin (with and without fentanyl), other synthetic opioids, and buprenorphine and methadone obtained illicitly. Not all patients with OUD are prescribed buprenorphine products within the program; some obtain methadone through providers at other substance use programs, and attend the program where the research occurred primarily for perinatal care. However, for the larger study from which a sub-analysis is herein presented and based on input from program providers who specifically desired to learn about access barriers affecting OUD patients receiving buprenorphine at that time, inclusion criteria for the larger study included that participants be prescribed buprenorphine products by program providers.

### Human subjects protection and ethics review

The full study protocol was reviewed by the Institutional Review Board (ethics review committee) at the regional hospital, which, at the time, reviewed all proposals for human subjects’ research conducted by researchers based at the institution that employs the authors and/or involving patients seen at the treatment program described.^4^ Due to the de-identified and qualitative nature of data collection (interviews and participant-observation) and because no aspect of the data collection would affect current pregnancies or future fertility, the study was determined exempt (Category 2) by the IRB. All participants in the larger study gave verbal consent to participate.

### Sampling strategy, recruitment, and data collection

Data were collected in late 2017 and early 2018 through a combination of opportunistic, convenience, and purposive sampling. The researchers had access to recruit participants onsite at the substance use treatment program all patients attended.

### Recruitment & eligibility

The researchers had access to eligible participants at the invitation of the program directors, who asked for the research to be conducted so that they could eventually learn more about their buprenorphine patients’ experiences seeking and receiving care. All potentially eligible participants were approached with information about the study and participants identified as eligible based on the inclusion criteria (patient sample: over 18, diagnosed with perinatal OUD, treated with buprenorphine) were specifically invited to participate in semi-structured interviews and/or a focus group. All patients attending the program during the study period were also informed and consented about participant-observation ongoing in the clinic and offered the option to decline to be observed with no negative consequences for their receipt of care.

### Data collection

The first two authors conducted all data collection. Both are trained in social science research methods including reflexivity and triangulation of multiple data sources [43, 44]. Data sources for the patient sample consisted of semi-structured interviews (*n* = 31); a focus group conducted during one session of an existing group prenatal visit organized for patients in the program (*n* = 2; only two patients attended the group prenatal visit on the date approved for conducting the focus group concurrently and both agreed to participate); and participant-observation of patient-provider and provider-provider interactions in the clinical setting (during 2 months of the overall study period). Patient interviews lasted approximately 25 min up to an hour; the focus group lasted for an hour; participant-observation took place in the clinic 2 days per week throughout the study period (October 2017 through February 2018). In the larger study, providers were interviewed as key informants (*n* = 10); those findings are not included here. For the subset analysis presented here, only data collected from the overall patient sample, representing the patient participants (*n* = 27) were re-analyzed (Table 1).

**Table 1:** Data sources

Data sources	Sampling strategy	Sample size ***n*** =	Nested sample	Included in subset analysis
**Patient interviews, prenatal**	Opportunistic, convenience, purposive	18	x	x
**Patient interviews, postpartum**	Opportunistic, convenience, purposive	11	x	x
**Focus group**	Opportunistic, convenience, purposive	2	x	x
**Participant-observation**	Opportunistic, convenience, purposive	4 months	x	x
**Patient total** ***n = 27*** (participant-observation represented a larger number of patients seen in clinic during study period)				
*Provider interviews*	*Purposive, key informant*	*10*		

Questions and prompts on the interview guides (Additional file 1) were informed by a review of existing literature on perinatal substance use treatment experiences, and by participant-observation. The guides included questions about participants’ experiences with and perceptions of the perinatal substance use treatment program; delivery/birth experiences (for postpartum interviews); if they felt the program had helped them achieve goals related to treatment; and if they felt the program had adequately prepared them for what would happen at delivery (postpartum interviews). The same topics guided observations during participant-observation; though observations were open-ended as and intended to inform the more formal data collection. Participant-observation was documented in de-identified fieldnotes journals, as is standard in ethnographic and anthropological fieldwork [43, 44].

Participants were interviewed one-on-one after appointments or during a break during steps of an appointment. In addition, patients in the program who met weekly in an existing group prenatal visit, typically led by one of the buprenorphine prescribers and a substance use counselor, were screened and all were found to be eligible for the interview portion of the study. Group members were asked if they were interested in having one session of their weekly prenatal group conducted as a focus group, discussing the same topics in the prenatal interview guide. All in attendance during the designated week consented, constituting a further nested opportunistic, convenience, and purposive sample. One participant in the focus group also participated in a one-on-one interview during the study period – however, that participant discussed different themes during her one-on-one interview as compared to during the triangulation focus group, her contributions were coded and analyzed separately from the two sessions. All interview and focus group participants received a Babies ‘R’ Us™ gift card worth $10.00 (U.S.) to thank them for taking time to share their perspectives.

### Overall data analysis

We analyzed demographic and quantitative data from demographic/ice-breaker questions asked at the beginning of each interview, to generate descriptive statistics about the participant sample in Excel (Table 2). We used modified Grounded Theory [45, 46] to analyze qualitative data from interview transcripts and fieldnotes. Interviews were transcribed by an outside firm; the first two authors transcribed our own fieldnotes. All patient interview and focus group transcripts and participant-observation fieldnotes were fully de-identified prior to hand-coding, with pseudonyms chosen by the participant (including nicknames, initials, and their preferred spelling) inserted to replace the spelling of any patient or provider names – these pseudonyms appear throughout this article. No member of the research team had access to participants’ real names during data analysis.

**Table 2:** Participant characteristics (*n* = 27)

Characteristic	Value
**Age**	Median 28 years old Mean 28 years old Range 22–37 years old (wider in participant-observation sample)
**Race/ethnicity**	26 White; 1 Black
**Number of children prior to current pregnancy**	Average 1–2 Range 0–4
**Gestation when entered program** (known for *n* = 9)	Average 17–18 weeks
**Pregnancy-related Medicaid coverage**	< 90% of all patients in comprehensive program

Modified Grounded Theory is an approach to analyzing ethnographic and qualitative research that allows ideas, themes, and relationships between themes to emerge organically from within the data, rather than entering into data collection and analysis with a specific hypothesis to be tested [45]. In standard modified Grounded Theory technique, the first two authors developed a codebook based on a combination of salient concepts from existing literature and early themes evident from open-coding of their fieldnotes from participant-observation. They each coded the first three interview transcripts and iteratively discussed emerging themes; revising and finalizing the codebook in the process. The first two authors then hand-coded all interviews and field notes, meeting regularly to compare the coding and continually refining the codebook until reaching full inter-rater agreement. With any changes to the codebook, transcripts that had already been coded were re-coded. After completing coding, the first two authors constructed a theoretical schema or concept map from the emergent themes to visually depict and further interpret relationships between the recurring topics and emic codes used by participants to describe their experiences.

### Subset analysis

The overall analysis of patients’ responses to interview and focus group questions about their experiences with perinatal substance use treatment; any obstacles or barriers; what they appreciated or found difficult about the program; their birth/delivery experiences (if the interview was conducted postpartum); and anything else they wanted to tell the authors revealed overall themes from the larger study that are presented elsewhere [10]. Among these, many participants in the comprehensive perinatal substance use treatment program – 11 of 27 – mentioned specific concerns about outside agencies’ involvement in their pregnancies and parenting resulting from their substance use. This theme from the larger study is the focus of the subset analysis presented here. Re-examining interview and focus group transcripts, and participant-observation fieldnotes from all participants (not just those who mentioned Social Services Involvement), the first two authors designed a subset analysis to more closely evaluate how patients’ perceptions of Social Services (child protective services) seemed to influence their feelings about engagement with perinatal substance use treatment. Theoretical findings were then discussed with other authors to negotiate a full analysis of findings with the full team of authors, including clinical providers who see perinatal patients with OUD diagnoses. We particularly sought to interpret subthemes about patients’ fears of Social Services involvement and the clinical implications of these for access to perinatal substance use treatment.

The subset analysis research design allowed for triangulation of key themes and concepts across several data sources: interview and focus group data, and participant-observation fieldnotes. Cross-comparisons of analysis between various data sources confirmed shared experiences, key points of agreement, and any points of departure.

## Results

In the larger study, 18 patient interviews took place prenatally and 11 post-partum; two patients participated in a focus group. Together this represented a nested sample with a total *n* of 27 participants participating in 31 interviews and/or one focus group. Several people participated in both a prenatal and postpartum interview; one person participated in a one-on-one interview and in the focus groups. Participant ages ranged from early twenties to late thirties. All but one participant was white. The majority of participants had children prior to the current pregnancy, also typical of the broader patient population in the program. Distances traveled to reach the program ranged from 15 min to 120 min, with an average travel time of 35 min. More than 90% of patients in the program rely on pregnancy-eligibility Medicaid for access to healthcare. A total of 27 patients participated in interviews and the focus group in the larger study; the subset analysis presented here includes data from all 27 patient participants.

Three interrelated subthemes emerged from the subset analysis and triangulation of interviews, a focus group, and participant-observation with all patient participants (*n* = 27): *Fears of Social Services Involvement; Preparation for Delivery;* and *Providers Addressing Fears.*

### Fears of social services involvement

Patients reported being afraid that Social Services would become involved after delivery as a result of their OUD, and treatment for it. They described having these fears before entering treatment. As one participant, “Billy,”^5^ said:

*… I was scared coming here, because I thought it’s gonna be immediate Social Services [involvement]. I [thought I] was never gonna see my child again… I never heard of this program, until the day I admitted I needed help, and that day was terrifying for me, because I’d had no idea what was gonna happen… people had told me* [seeking treatment would mean immediate removal]…

Women with OUD were fearful of the social and legal ramifications of accessing substance use treatment, internalizing and embodying messages they had heard from family, friends, peers, or other community members. These included a fear that by engaging in care, Social Services would find out they were receiving MAT -- which they believed would lead to automatic removal of their child as Billy referenced above.

Another participant added:

*I knew that my cord blood was gonna be tested, and I was convinced that, instantly, Social Services was gonna come in, that there was gonna be… – [that] I was gonna lose my kid.* (Grace^5^)

Here, Grace references the law in her state that governs healthcare provider and facility requirements to notify and/or report suspected or confirmed substance use exposure affecting newborns. Grace was aware that the delivering hospital routinely tests neonatal umbilical cord tissue for all patients whose charts reflect they have been on MAT during pregnancy. A positive presence of opioids, including prescribed medications such as buprenorphine products or other MAT modalities requires a notification to Social Services. Like Billy, Grace was fearful about how engaging with treatment would affect any ensuing interactions with Social Services. She held this fear despite the fact that this treatment is recommended by medical and obstetric associations, proven safe and effective, and the treatment approach to which her perinatal providers had encouraged her to adhere. She feared she could lose custody as a result of being on prescribed, evidence-based MAT. During participant-observation we heard similar fears expressed during many clinic interactions, in the form of questions asked of the PSE.

A participant in the focus group echoed Grace and Billy’s assumptions:

*I can see why a lot of people are nervous about Social Services. I’ve had a lot of people say like, ‘well since you’re you know—in the [*buprenorphine*] clinic, they’re going to automatically –Social Services is automatically going to come to see you.’*

Patients were unaware or unclear on the specific factors that would generate a notification to Social Services. This confusion only enhanced what they described as their greatest fear: losing custody of their child -- not as a result of opioid use, but as a result of seeking treatment for OUD. As we learned in interviews and through participant-observation in the clinic, participants reported they often felt powerless to change this outcome and were fearful of how engaging in treatment would influence retaining custody.

### Preparation for delivery

Many participants reported having been afraid of Social Services involvement at the time they entered comprehensive treatment. Yet many also reported in interviews and informally during participant-observation that providers helped prepare them for that potential future involvement once they delivered, including explaining how their baby would be monitored and evaluated for NOWS. As Grace said, “*… the [*Perinatal Substance Educator*] walked me through, ‘*this is how [it’s gonna happen]… how the steps would play out*…” it went exactly how she said it would.”*

Ashley^5^, concurred, saying, “*the [*PSE*] told me that Social Services might get involved… they prepared me for that exactly.*” From our observations in the clinical setting, we also noted how both medical and behavioral providers on the team offered all patients a “warm hand-off,” [7] to the PSE.^6^ During bimonthly or more frequent appointments, perinatal OUD patients were routinely offered opportunities to meet with the PSE to discuss what would occur at the time of delivery. These consultations were integrated into regular appointments with prenatal and behavioral health providers.

Participants described the role the PSE and other providers played in helping them to create a plan and prepare for Social Services involvement after the delivery of their child. By creating a plan together with the PSE and other providers, participants reported they could take steps to mitigate the likelihood of Social Services involvement, thereby supporting and empowering them to reduce their fears about seeking perinatal substance use treatment. “K.K.”^5^ stated:

*My plan is set up for Social Services prevention. I don’t currently have Social Services involved in my life, but considering my [*substance use*] history and the fact that I’m on [*MAT*],[*I know*] Social Services is gonna come visit me at the hospital. So, it’s taking steps to prevent that and to help me with legal things that I have going on right now*.

The plan K.K. described was one she developed with the PSE. Participants such as K.K. reported increased understanding of how factors such as their substance use history, being on MAT, and being pregnant, could influence their potential involvement with Social Services following delivery. In interviews, many people attributed their increased understanding to having met with the PSE.

### Providers addressing fears

While acknowledging that hearing about Social Services notification and potential implications could be difficult, participants reported they appreciated knowing how Social Services might become involved and what to expect after delivery. Patients also appreciated the preparation and information about how their newborn would be treated in the clinical setting and how a new parent could identify symptoms of NAS in their newborn. Overall, participants described interactions with providers in the comprehensive program addressing their fears. As Morgan^5^ said:

*Yeah. I knew how he* [her baby] *would be scored [*referring to Finnegan scoring*] and what was gonna happen with him and stuff. And thankfully, he didn’t have to go through everything that they had told me would happen if he was [*identified as having symptoms of] *NAS when he born. But yeah, I was aware of everything that was gonna happen. I think it was a little easier than what I was expecting.*

Sarah^5^ concurred:

*[*I appreciated*] the information, how helpful they are, how open they are to giving you all the pros and cons. They’re very open about like this is what’s gonna happen when you get to the hospital. ‘They’re gonna scale the child on the Finnegan Scale.’ They’re very informational. Sometimes it’s kinda scary to hear all that, but I liked being prepared.*

Similarly, B.^5^ appreciated the knowledge about what Social Services involvement could look like after she delivered. She compared how the comprehensive program prepared her in the current pregnancy; compared to an earlier pregnancy when she was on methadone and not enrolled in a comprehensive program:

*Yeah, oh yeah. [*I felt prepared*] from the groups – yeah – from the groups and girls that had experienced it before us. What Social Services would say in [the hospital]. Because I knew my daughter – I went through it with my daughter [*on methadone at that time*], but that was like seven years ago. I know –* [the PSE*] told us, so much stuff has changed in the hospital since then that I didn’t know what to expect going in there. I didn’t know what the hell [*to expect*] Because [*people I talked to in the community previously*] kind of scared you telling you about Social Services getting in[volved] and all that stuff – it’s kind of scary.*

Other participants described how having supportive and non-judgmental caregivers made them feel they had someone on their side and helped reduce their anxiety about potential involvement with Social Services: “*… You’re also able to actually tell Social Services about it…because they are treating it like, ‘*You have a caregiver while you’re on the Suboxone™?*’ [so] it doesn’t mess me up.”* (Skye^5^)

What Skye alludes to here is a widespread understanding among OUD patients, and providers, that many low-income people with OUD or a history of substance use may have previously obtained buprenorphine illicitly, often to self-treat their own substance use disorder or to avoid withdrawals from opioids. Being able to receive a licit prescription for buprenorphine and understanding from the PSE how to interact with Social Services at the time of their child’s delivery was likely to go, removed much of the fear participants had lived with.

Skye’s quote also demonstrates that participants learned from working with a comprehensive team of providers that receiving prescribed MAT would not necessarily hinder their interactions with Social Services but instead was a way that patients could demonstrate they were accessing formal treatment for their OUD (as opposed to obtaining illicit buprenorphine, which they may have previously done to self-treat OUD). Participants articulated they felt prepared to be more open with Social Services about being on buprenorphine following a required notification because a licensed provider prescribed the medication. Preparatory conversations with providers helped allay participants’ fears of Social Services.

By alleviating these fears participants also reported being more able to enjoy the prospect and experience of childbirth:

*And [*the PSE*] was just like, ‘*No, that’s – We don’t – We don’t wanna take your kid. We want your child to stay with you*’ And it was nice to be told that, no, you don’t have to worry. I was able to relax more and enjoy being there and having the process of having a baby, let my husband enjoy his first child, that kind of thing.* (Grace)

In participant-observation, we noticed patients who came into the room anxious about child removal and Social Services involvement became visibly more relaxed while talking to the PSE. Having a chance to ask questions, hear what would happen at the hospital when they delivered, and even discuss past experiences with child removal seemed to alleviate fears and increase trust in the provider team. In response to open-ended questions about the experience of seeking perinatal substance use treatment, no participants reported *not* appreciating having had the opportunity to talk to the PSE. During participant-observation we did not observe any patients decline to meet with the PSE.

## Discussion

The almost entirely white ethnic/racial make-up of the overall sample was representative of the largely rural, Southeastern Appalachian region in which the research was conducted and of the clinic’s patient population as a whole. This sample is also consistent with existing literature documenting reduced access to OUD treatment for cisgendered women of color and other marginalized populations.

Within this rural Southern Appalachian region, we determined that patients in a comprehensive perinatal substance use treatment program often internalized and embodied fears and stigma about MAT and its potential consequences. These fears resulted largely from structural and policy factors intersecting with substance use and MAT stigma, rather than from concerns about clinical aspects of care. Participants became aware of the structural and policy implications of their OUD and use of MAT through information that trickled down from local bureaucracies and authority figures and healthcare institutions, often passed along by friends, family, other community members, or as experienced directly in earlier pregnancies. Thus, they largely entered substance use treatment in a current pregnancy already aware of the prospect of Social Services involvement. Fears were compounded due to patients’ dually scrutinized and stigmatized social identities as pregnant people with OUD, and, for the vast majority of patients in the program, also being seen as poor people reliant on Medicaid for perinatal care. Our study reconfirms the conclusions of earlier research that suggested providers should be aware that perinatal OUD patients enter treatment likely fearful of child removal.

Participants entered treatment confused and unclear about what Social Services involvement would entail, but generally assumed their infant would be removed from their care soon after delivery – not for active drug use, but for having sought substance use *treatment*. As identified in the larger study of which this is a subset, widespread societal surveillance of low-income people’s pregnancies, especially in the context of substance use and treatment, increases stigmatization of this population’s childbearing and reproductive decision-making. Such dual stigmatization of pregnancy and substance use/treatment in the context of the care our participants sought likely contributed to their intense concern about Social Services involvement, shaping some participants’ assumptions about what would occur. These assumptions persisted until they actually met with the PSE and received information about what to expect at the time of delivery.

Fellow perinatal substance use treatment researchers recommend explicit education about child protective services for both patients and providers. Our findings demonstrate the value of the PSE as part of the interdisciplinary provider team, enhancing patients’ understandings of what to expect at delivery. Participants’ comments about the PSE and the information they received reflect the important role perinatal substance use treatment providers can play in supporting patients to identify, acknowledge, and address fears that go beyond clinical aspects of care.

Though an accurate, supportive conversation about Social Services involvement post-delivery may be challenging to integrate into the clinical setting given provider time constraints, participants clearly appreciated when providers talked with them and helped prepare them for what would occur post-delivery. Answering the call of earlier studies for more research on how CAPTA laws and mandatory reporting affect relationships with providers, this study underscores the importance of providers acknowledging patients’ fears and patients feeling they can trust providers, in the context of such laws. Our findings suggest obstetrical providers should know how their specific local settings – state, county, specific healthcare facilities, etc. – interpret current CAPTA laws and mandatory Social Services reporting or notification requirements. Providers should ensure their patients have the opportunity to hear and talk about the potential for Social Services involvement, in order to address and discuss any fears. This role does not have to filled by a physician; it can be served by another person in the office or community with the ability to share this knowledge and engage patients in planning. As we observed in the interdisciplinary integrated program where we conducted this study, a PSE can have nearly any background or credentials; key components of their role include (1) understanding clinical aspects of the outpatient and inpatient OB delivery teams; (2) understanding the delivering hospital’s inpatient social work and neonatal care teams; (3) understanding local social services, and most importantly, (4) having support from their employer to convey all of this knowledge to patients and engage with overlapping care teams in an ongoing way.

### Limitations

The data collected only included perspectives of perinatal OUD patients that came to treatment; we did not capture the experiences of people not engaged in treatment, nor do we know the perspectives of perinatal OUD patients or perinatal patients with other SUDs participating in non-comprehensive programs. Moreover, this study was designed to exclusively sample perinatal OUD patients treated with buprenorphine products prescribed in one comprehensive program in the Southern Appalachian U.S. Thus, this research does not speak to the experiences of perinatal patients in this region receiving methadone during the perinatal period, nor of those with other SUDs – both groups would be equally subject to CAPTA laws and notification requirements. Nevertheless, this study reveals more than has been documented previously about what accessing MAT means to an at-risk, rural population, particularly in the context of Social Services notification requirements -- and how providers can help address patient fears of bureaucratic surveillance.

## Conclusion

Perinatal patients are aware and afraid of Social Services involvement when seeking OUD treatment. This study contributes to filling what is otherwise a gap in the literature about how providers can address such fears. Perinatal patients appreciated the honest conversations providers facilitated about Social Services notification requirements where they live, and what to expect at delivery. Creating the space for such conversations -- by *initiating* them -- and ensuring related expertise on the clinical team -- can serve to build trust between patients and providers. Providers talking openly about Social Services involvement, otherwise ‘the elephant in the room’ as found in earlier research, made it easier to confront and address such fears.

We recommend perinatal substance use treatment programs and all providers screening patients capable of pregnancy for substance use disorders integrate a PSE in their care team and offer specific information about local notification requirements and state-level interpretation of CAPTA law (or equivalent). Perinatal programs should become aware of their local hospital’s notification process, as well as how surrounding counties and the state interpret CAPTA laws, and have a designated team member available to talk with patients. This role does not have to be filled by an MAT prescriber or behavioral health clinician who might be required to make a CAPTA notification, but ideally should be another person in the office or community with the ability to address the elephant in the room -- engaging patients in important and valued conversations about fears of Social Services involvement. This should address and help allay the fears of pregnant people with OUD, and enhance trust in their providers.

## Supplementary Information

**Additional file 1.**

## Data Availability

Given the nature of the interview questions, the characteristics of the study participants connected to this program which is the only one of its kind in a large geographic region, and the ethics committee-approved consent process which included the participants receiving assurance that no-one other than study team members would have access to transcripts of their interviews, we are not allowed to make the data available. Doing so would violate our ethics approval.

## Abbreviations

CAPTA: Child Abuse Prevention and Treatment Act
MAT: Medication-assisted treatment
NAS: Neonatal abstinence syndrome
NICU: Neonatal intensive care unit
NOWS: Neonatal opioid withdrawal syndrome
OUD: Opioid use disorder
PSE: Perinatal substance use exposure educator
SUD: Substance use disorder

## Footnotes

1 Fifteen states are designated as the U.S. South by the U.S. Census Bureau, including all of Southern Appalachia, https://www2.census.gov/geo/pdfs/maps-data/maps/reference/us_regdiv.pdf.

2 Finnegan scores are assigned by observing a newborn’s level of alertness, feeding patterns, muscle tone, crying, level of perceived agitation, etc.

3 This information was shared in personal correspondence from an employee with the North Carolina Department of Health and Human Services.

4 The authors are not allowed to name the specific institutional review board/ethics committee. It was, at the time of the study, the only such ethics committee that reviewed studies for all research conducted at the study site; the ethics committee is based at a hospital where all study participants receive care; thus naming it would risk making the study site and/or participants (who have unique characteristics and attend the only program of its kind in a large geographic region), identifiable. Under the authors’ institutional review board ethics committee approval for this study, both the site and the participants must be kept de-identified. Naming the specific ethics review committee would violate our ethics approval.

5 Note: All participant names/nicknames/initials are pseudonyms they chose for themselves, and spelled as they indicated.

6 Warm hand-offs are a recommended strategy in behavioral health and substance use treatment overall that emphasizes direct referrals, preferably with face to face introductions for a patient to another provider or participating team member [47].
