# Supplementary material for: “The elephant in the room;” a qualitative study of perinatal fears in opioid use disorder treatment in Southern Appalachia
Source: BMC Pregnancy Childbirth. 2021 Feb 17;21:143. doi: 10.1186/s12884-021-03596-w (PMC7890815; doi:10.1186/s12884-021-03596-w)
Supplement: Supplementary file 1 — Additional file 1. [file 12884_2021_3596_MOESM1_ESM.pdf]

☐ *Verbally consented to participate?* ☐ *Pseudonym?*
☐ *To be recorded?* ☐ *Attends group visits?*

PRENATAL

1) **How did you find out about** [the combined perinatal and substance use treatment clinic]?

2) **What has** [coming to the clinic] **been like for you?**

3) a. **Is there anything you specifically appreciate about** [the combined perinatal and substance use treatment clinic]? *(Probe/prompt - What* about *the program, works for you?)*
b. **Is there anything that is particularly hard about [**the combined perinatal and substance use treatment clinic] **about the program**? *(Probe/prompt - Are there any barriers to substance use treatment during pregnancy, for you?)*

4) *(If participates in prenatal group visits)* – **What are group visits like for you?** How do they fit into your overall treatment and prenatal care?
5) Is there **anything that would be helpful to you in your care that is *not* part of** [the combined perinatal and substance use treatment clinic] so far?
(Probe/prompt -- Specific services you'd want to be to connected to?)

6) What are your **goals for combined substance use treatment/prenatal care?** Do you feel [the combined perinatal and substance use treatment clinic] **is helping** [you achieve those goals]?
(Clarify - How so?/Why or why not?)

7) **Anything else you want to tell me** about your experiences with substance use treatment and prenatal care?

POST-PARTUM

All relevant questions from above if not interviewed prenatally (but past tense if re: pregnancy)
and

- Any **services you feel would be helpful now that are *not* part of** [the combined perinatal and substance use treatment clinic]?

(Probe/prompt -- ***Postpartum services*** *you'd want to be to connected to?)*

- Please tell me about your **birth/delivery experience**

(start open-ended; check off items below if mentioned or probe if not mentioned and if seems appropriate)
  - *Where was baby born?*
  - *How treated in different areas of the hospital? (How would you* like *to have been treated?)*
  - *Anything you would have liked to have been different? (delivery, or right after baby was born?)*
- Did [the combined perinatal and substance use treatment clinic] **prepare you enough for what would happen at the hospital?**
(Clarify -- How so, or not?)

- **Anything else you want to tell me** about your experiences with substance use treatment, and pregnancy and birthing care?
